# Infectious Complications and Safety Outcomes in Cholangioscopy: A Systematic Review and Meta-Analysis

**DOI:** 10.1055/a-2877-9645

**Published:** 2026-06-10

**Authors:** Shivangini Duggal, Akshay Sharma, Vishali Moond, Simran Joshi, Mehak Sachdeva, Nirav Thosani, Prateek Harne

**Affiliations:** 1 Department of Internal Medicine 37316 Texas Tech University Health Sciences Center El PasoEl PasoTexasUnited States; 2 Department of Internal Medicine 536507 Luminis Health Anne Arundel Medical CenterAnnapolisMarylandUnited States; 3 Department of Gastroenterology 5631 West Virginia UniversityMorgantownWest VirginiaUnited States; 4 Department of Internal MedicineBridgeport Hospital, Yale New Haven HealthBridgeportConnecticutUnited States; 5 Department of Internal MedicineUniversity of Central Florida/HCA Florida Ocala HospitalOrlandoFloridaUnited States; 6 Department of Interventional Gastroenterology 12340 The University of Texas Health Science Center at HoustonHoustonTexasUnited States; 7 Department of Interventional Gastroenterology 205980 DHR HealthEdinburgTexasUnited States

**Keywords:** cholangioscopy, bacteremia, antibiotic prophylaxis, adverse events, choledocholithiasis, SpyGlass

## Abstract

**Background** Single-operator cholangioscopy allows direct visualization and intervention for complex biliary disease but has been associated with higher infectious adverse events compared with standard endoscopic retrograde cholangiopancreatography. Evidence guiding antibiotic prophylaxis specific to cholangioscopy remains limited. We performed a systematic review and meta-analysis to quantify infectious complications following cholangioscopy.

**Methods** PubMed, Embase, Scopus, Google Scholar, and the Cochrane Library were searched through March 2025. Studies reporting post-procedural bacteremia after cholangioscopy were included. Random-effects meta-analyses estimated pooled rates of bacteremia and secondary outcomes, including cholangitis, pancreatitis, technical success, clinical success, and adverse events. Sensitivity analyses and univariable meta-regression were conducted to assess heterogeneity. Publication bias was evaluated using funnel plots and Egger’s test.

**Results** Twelve studies comprising 4413 cholangioscopy procedures were included. The pooled bacteremia rate was 6.68% (95% confidence interval [CI], 1.96–20.44; *I* ^2^ = 95.5%). Pooled rates of cholangitis and pancreatitis were 4.59% (95% CI, 2.98–7.00; *I* ^2^ = 60.0%) and 3.54% (95% CI, 1.20–10.00; *I* ^2^ = 89.0%), respectively. Technical and clinical success rates were high at 97.21% and 87.13%. Sensitivity analyses demonstrated persistent heterogeneity across study design, indication, and procedure type. Meta-regression identified study design as a significant moderator, with prospective studies reporting higher bacteremia rates ( *p* = 0.018), explaining 43.8% of the between-study variance.

**Conclusions** Cholangioscopy is highly effective with a low rate of severe complications. Infectious adverse events occur in 6.7% of cases. Use of routine antibiotic prophylaxis remains uncertain; targeted preprocedural prophylaxis may be considered in high-risk patients. Prospective studies are needed to standardize preventive strategies.

## 1 Introduction

The use of choledochoscope/cholangioscope for the examination of the biliary tree during endoscopic retrograde cholangiopancreatography (ERCP) (traditional) or alone (direct peroral) is becoming a widespread technique as it allows direct endoscopic visualization of the biliary system. This enables the treatment of choledocholithiasis, achieving selective biliary access, visualization of biliary pathologies, and collection of biopsy samples for histological analysis of biliary strictures or masses. 1 2

However, single operator cholangioscopy (SOC) is associated with a higher risk of adverse events (AEs) compared to ERCP alone. Especially for postprocedural cholangitis, multiple studies have reported postprocedural cholangitis rates ranging from 1% to 12.2% with SOC, 3 4 5 6 7 8 9 10 11 12 in contrast to 0.5% to 3% with ERCP alone. 13 14 15 Although small case series have suggested that these infectious complications may be preventable with the use of preprocedural antibiotics, 3 7 the approach to prophylactic antibiotic use has evolved over time. Initially, antibiotics were routinely administered before ERCP; however, the 2015 guidelines from the American Society for Gastrointestinal Endoscopy (ASGE) recommended limiting prophylactic antibiotics to cases involving incomplete biliary drainage, based on comprehensive literature reviews and meta-analyses. 16 The European Society of Gastrointestinal Endoscopy offers similar guidance without SOC-specific recommendations. 15 Because SOC involves prolonged biliary irrigation and often incomplete drainage, extrapolating ERCP-based guidance may underestimate infection risk.

Currently, a knowledge gap exists in clinical guidance when it comes to administering antibiotics for SOC procedures, necessitating further data on infectious complications such as bacteremia and cholangitis. Our study aims to address this gap by conducting a meta-analysis of existing literature to evaluate the rates of infectious complications, identify associated risk factors, and assess the role of periprocedural antibiotic use in SOC.

## 2 Materials and Methods

### 2.1 Study Design

This systematic review and meta-analysis was conducted in accordance with the Preferred Reporting Items for Systematic Reviews and Meta-Analyses (PRISMA) guidelines and the PRISMA Extension Statement for Network Meta-analyses. The objective was to evaluate the pooled rates of bacteremia and other infectious outcomes associated with SOC procedures. A detailed PRISMA checklist is provided in Supplementary **Table 1** .

### 2.2 Search Strategy

A comprehensive literature search was performed across multiple databases, including PubMed, Embase, Scopus, Google Scholar, and the Cochrane Library, covering all articles published up to March 2025. The search strategy combined terms related to “cholangioscopy,” “SpyGlass,” “single-operator cholangioscopy,” “bacteremia,” “cholangitis,” “infection,” and “AEs.” The full search strategy is available in Supplementary **Table 2** . Two reviewers (S.D. and A.S.) independently screened the titles and abstracts for relevance, followed by full-text review. Discrepancies were resolved through discussion and consensus or through consultation with a third reviewer (P.H.). Additionally, references from selected articles and related systematic and narrative reviews were manually examined to identify further relevant studies.

### 2.3 Study Selection

Studies were included if they reported outcomes of patients undergoing cholangioscopy procedures, specifically documenting the rate of postprocedural bacteremia. Both diagnostic and therapeutic cholangioscopy procedures (e.g., biopsies, laser lithotripsy) were eligible. Inclusion criteria encompassed prospective studies, retrospective studies, registry analyses, and clinical trials. Exclusion criteria included (1) case reports or case series with fewer than 10 patients, (2) studies without clear data on bacteremia rates, and (3) non-English language publications without accessible full-text translations. In instances of overlapping cohorts, the most recent or most comprehensive dataset was used.

### 2.4 Data Abstraction and Risk-of-Bias Assessment

Two reviewers (S.D. and A.S.) independently extracted data using a standardized data collection form. Extracted variables included study design, setting, sample size, technical and clinical success rates, use of antibiotic prophylaxis, bacteremia rates, cholangitis rates, pancreatitis rates, and other reported complications. Risk of bias was assessed independently by two reviewers using the Newcastle-Ottawa Scale for observational studies, evaluating selection, comparability, and outcome domains. Disagreements were resolved by consensus.

### 2.5 Definitions

Bacteremia was defined as a culture-positive bloodstream infection detected within 24 hours of SOC and encompassed episodes of sepsis or postprocedural fever requiring antibiotic therapy. Cholangitis was defined according to the Tokyo Guidelines when reported, requiring evidence of systemic inflammation together with cholestasis and/or imaging evidence of biliary obstruction. Infectious complications were analyzed as bacteremia, cholangitis, and pancreatitis reported separately as a procedure-related AE. All AEs were categorized according to the ASGE lexicon. Mild AEs were defined as those requiring unplanned medical consultation, extended observation, or hospitalization for ≤3 days without lasting disability (e.g., self-limited bleeding, transient fever, mild pancreatitis). Moderate AEs required active medical intervention or hospitalization for 4–10 days but resolved without long-term sequelae (e.g., post-ERCP pancreatitis requiring intravenous therapy or moderate cholangitis). Severe AEs included events necessitating intensive care unit admission, surgical or percutaneous intervention, hospitalization >10 days, or resulting in long-term disability, whereas fatal AEs referred to any procedure-related death.

### 2.6 Outcomes Assessed

The primary outcome of interest was the pooled rate of bacteremia following cholangioscopy. Secondary outcomes included pooled rates of technical success, clinical success, cholangitis, pancreatitis, and other AEs. Technical success was defined as successful visualization or intervention completion, while clinical success was defined as symptom resolution or complete ductal clearance, depending on the study’s context. Infectious outcomes were defined as per individual study criteria and included culture-proven bacteremia or clinically diagnosed postprocedural infection.

### 2.7 Statistical Analysis

Meta-analysis techniques were employed to calculate pooled estimates using the random-effects model as proposed by DerSimonian and Laird. In cases where zero events were recorded for a given outcome within a study, a continuity correction of 0.1 was applied as a standard approach to enable pooled analysis. Heterogeneity among study estimates was assessed using the Cochran Q test and *I* ^2^ statistics, with values indicating low (<30%), moderate (30–60%), substantial (61–75%), and considerable (>75%) heterogeneity. Publication bias was evaluated qualitatively through funnel plots and quantitatively using the Egger test when more than 10 studies were included. Meta-analyses were performed using R statistical software, version 4.5.2 (2025-10-31) (R Foundation for Statistical Computing, Vienna, Austria), employing random-effects models implemented in the meta package. All pooled estimates are reported with 95% confidence intervals (95% CIs). All *p* -values are two-sided, with statistical significance defined as *p* < 0.05. Descriptive statistics are presented as mean ± standard deviation (SD), or as median with interquartile range (IQR) or range, as appropriate. Additional statistics reported include the standard error (SE), *t* -statistic ( *t* ), and between-study variance (tau-squared, τ ^2^ ), the latter of which is displayed in forest plot figures.

## 3 Results

### 3.1 Search Strategies and Population Characteristics

From an initial total of 1402 search results, 1291 titles were screened, and 342 articles underwent full-length review. A total of 12 studies encompassing 4413 cholangioscopy-guided procedures were included in this meta-analysis, representing diverse geographical regions including the United States, Europe, India, and Japan. The schematic diagram of the study selection process is provided in Supplementary **Fig. 1A** .

### 3.2 Characteristics and Quality of Included Studies

Table 1 describes the population characteristics. Most patients were male (57.1%). The reported median ages across studies ranged from 28 years (IQR 20–70) to 72 years (IQR 42–90). Among studies reporting mean ages, the average age was 61.4 years (±17.1). Technical success was defined as successful deployment and positioning of the cholangioscope allowing direct visualization and instrument maneuvering to the targeted area of interest across all studies. Clinical success was defined as completion of the intended diagnostic or therapeutic objective, such as stone fragmentation and removal, targeted biopsy acquisition, or diagnostic clarification of biliary pathology.

**Table 1:** Summarizes the population characteristics across all studies.

Serial number	Study (Abbr)	Year	Total number of patients	Mean age (±SD) or median age (IQR) in years	Gender (males %)
1	Othman et al.	2016	57	58.3 ± 18.4	21 (37%)
2	Pereira et al.	2022	85	65 (59–72)	56 (65.90%)
3	Thosani et al.	2016	72	66.3 ± 11.1	44 (69.80%)
4	Chandan et al.	2024	1743	–	–
5	Brewer Gutierrez et al.	2018	407	63.3 ± 19	161 (39.60%)
6	Bhandari et al.	2016	34	28 (20–70)	15 (44.11%)
7	Alexandrino et al.	2022	94	71 (32–97)	39 (41.49%)
8	Arnelo et al.	2015	47	40 (22–70)	33 (70.00%)
9	Hüsing-Kabar et al.	2017	26	54.5 (25–75)	12 (46.20%)
10	Canena et al.	2019	17	72 (42–90)	11 (65.00%)
11	Gustafsson et al.	2023	1605	57.9 ± 17.4	915 (57.00%)
12	Minami et al.	2021	183	69.8 (35–93)	120 (65.57%)

The 12 included studies comprised a mix of prospective ( *n* = 6) and retrospective ( *n* = 6) designs. All of the included studies, except that by Chandan et al., had clear information reported on the technical success, clinical success, and AE rates. All included studies are original manuscripts. Prospective studies ranged from observational single-center cohorts ( *n* = 3) to multicenter evaluations ( *n* = 3), while retrospective analyses included both institutional and database-driven studies, such as those utilizing the FDA MAUDE and GallRiks registries. Six of the included studies were of high quality, five studies were of medium quality, and one study was of low quality. The detailed quality assessment is summarized in Supplementary **Table 3** .

### 3.3 Meta-analysis Outcomes

A total of 12 studies were included in the quantitative synthesis. The pooled rate of bacteremia, which was the primary outcome of interest, was 6.68% (95% CI, 1.96–20.44, *I* ^2^ = 95.5%). Subgroup analysis stratified by study design demonstrated significantly higher bacteremia rates in prospective studies (22.1%, 95% CI 6.1–55.2; *I* ^2^ = 84.9%) compared with retrospective studies (2.0%, 95% CI 0.4–10.6; *I* ^2^ = 94.2%), with a significant difference between groups ( *p* = 0.003) (Supplementary **Fig. 1B** ). The pooled incidence of cholangitis was 4.59% (95% CI, 2.98–7.00, *I* ^2^ = 60.0%). Postprocedural pancreatitis occurred at a pooled rate of 3.54% (95% CI, 1.20–10.00, *I* ^2^ = 89.0%). Five perforation events were identified in the patient cohorts (Arnelo et al., *n* = 2; Minami et al., *n* = 1; Pereira et al., *n* = 1; Bhandari et al., *n* = 1). Procedural performance outcomes demonstrated high success rates, with a pooled technical success rate of 97.21% (95% CI, 94.05–98.71, *I* ^2^ = 50.0%) and a pooled clinical success rate of 87.13% (95% CI, 75.57–93.68, *I* ^2^ = 82.0%).

Notably, several studies reported significantly elevated bacteremia rates when preprocedural antibiotics were omitted or when biopsies were performed (e.g., Othman et al. and Thosani et al.). Across studies, biopsy was frequently performed (e.g., 14 biopsies in Othman et al., 89 in Minami et al.) and was associated with higher rates of bacteremia in selected cohorts. Lithotripsy procedures were included in eight studies, primarily utilizing electrohydraulic or laser modalities; however, only two studies (e.g., Alexandrino et al., Canena et al.) reported post-lithotripsy infection rates, without a clear causal linkage.

Individual study rates of bacteremia varied substantially across all studies. Thosani et al. (2016) reported the highest bacteremia rate at 27.8% (20/72), including 13.9% sustained bacteremia attributed specifically to SOC, despite postprocedure antibiotic prophylaxis. Canena et al. (2019) observed bacteremia in 4 of 17 patients (23.5%), all of whom had received intravenous ciprofloxacin. Alexandrino et al. (2022) reported bacteremia in 22.3% (21/94) despite all patients receiving intravenous antibiotics. Othman et al. (2016) found a bacteremia rate of 8.8% (5/57); all patients were antibiotic naïve, and blood cultures were obtained preprocedure and postprocedure to confirm positivity. Hüsing-Kabar et al. (2017) reported a notably high rate of 69.6% (16/23) bile culture positivity in liver transplant recipients undergoing ERCP with SOC. Minami et al. (2021) noted bacteremia in 13.7% (25/183), with significantly fewer febrile events in patients who received prophylactic antibiotics. Bhandari et al. (2016), focusing on SOC-guided laser lithotripsy for Mirizzi syndrome and cystic duct stones, observed a 5.9% (2/34) incidence of bacteremia. Gustafsson et al. (2023), in a national registry-based cohort study of 1605 patients from Sweden, documented 3.4% (55/1605) bacteremia cases. Arnelo et al. (2015), evaluating SOC in PSC patients, reported one bacteremia case (0.02%) among 47 patients, all of whom received prophylactic antibiotics. Brewer Gutierrez et al. (2018) reported only one case (0.2%) of bacteremia among 407 patients undergoing digital SOC for difficult stones, with a 92% prophylaxis rate. Pereira et al. (2022) reported only one bacteremia case (1.2%) in 85 patients undergoing D-SOC for biliary stricture evaluation. Finally, the MAUDE database analysis by Chandan et al. (2024) identified six bacteremia cases among 1743 reports (~0.003%).

AEs classified according to the ASGE lexicon were also analyzed. The pooled rate of mild AEs was highest 7.51% (95% CI, 3.50–15.40, *I* ^2^ = 90%). Moderate AEs occurred at a pooled rate of 4.58% (95% CI, 2.15–9.50, *I* ^2^ = 90%) and severe AEs were uncommon, with a pooled rate of 0.81% (95% CI, 0.33–2.00, *I* ^2^ = 60%). Fatal AEs were rare, occurring at a pooled rate of 0.40% (95% CI, 0.17–0.97, *I* ^2^ = 10%). Follow-up durations ranged from 24 hours to 64 months. Several studies incorporated blood culture collection protocols preprocedure and postprocedure (e.g., Thosani et al., Othman et al.), contributing to bacteremia detection. A comprehensive summary of study outcomes is provided in Table 2 and Fig. 1 .

**Table 2:** Displays the results across each outcome for this meta-analysis.

Outcome	*N* (studies)	Pooled rate (%)	95% CI	*I*^2^ (%)
**Bacteremia**	12	6.68	1.96–20.44	95.5
**Cholangitis**	10	4.59	2.98–7.00	60.0
**Pancreatitis**	10	3.54	1.20–10.00	89.0
**Technical success**	10	97.21	94.05–98.71	50.0
**Clinical success**	9	87.13	75.57–93.68	82.0
**Adverse events (ASGE Lexicon)**				
**Mild adverse events**	12	7.51	3.50–15.40	90
**Moderate adverse events**	12	4.58	2.15–9.50	90
**Severe adverse events**	12	0.81	0.33–2.00	60
**Fatal adverse events**	12	0.40	0.17–0.97	10

**Fig. 1a:**
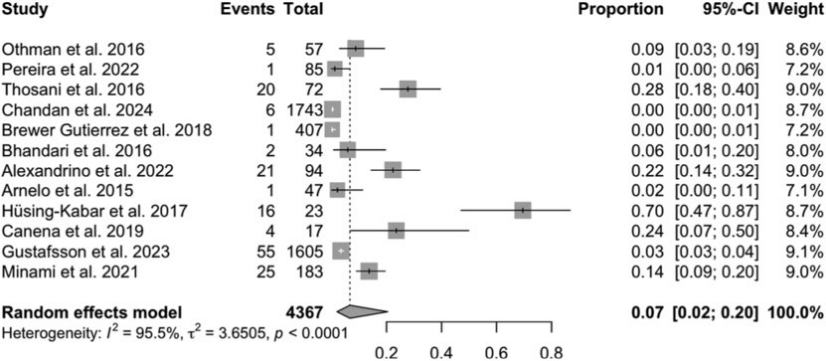
Forest plots summarizing pooled event rates and 95% confidence intervals for infectious adverse events following peroral cholangioscopy across the included studies. (I) Bacteremia (pooled event rate, 6.68%).

**Fig. 1b:**
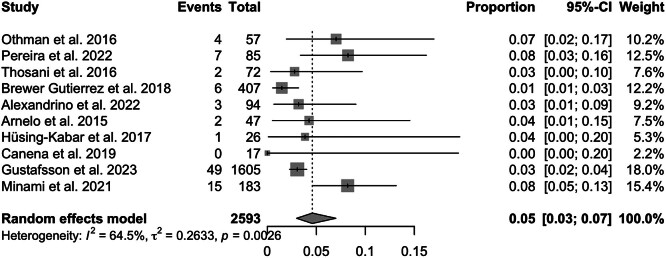
Forest plots summarizing pooled event rates and 95% confidence intervals for infectious adverse events following peroral cholangioscopy across the included studies. (II) Cholangitis (pooled event rate, 4.59%).

**Fig. 1c:**
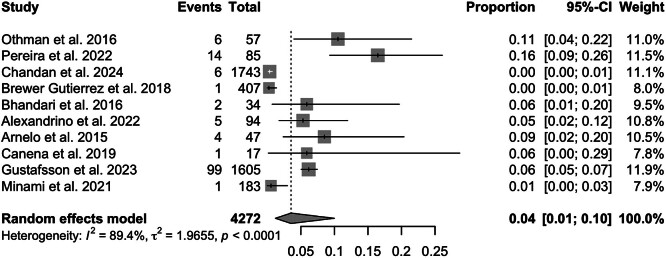
Forest plots summarizing pooled event rates and 95% confidence intervals for infectious adverse events following peroral cholangioscopy across the included studies. (III) Pancreatitis (pooled event rate, 3.54%).

**Fig. 1d:**
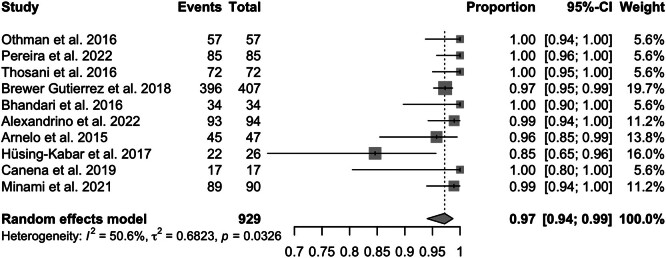
Forest plots summarizing pooled event rates and 95% confidence intervals for infectious adverse events following peroral cholangioscopy across the included studies. (IV) Technical success rate (pooled event rate, 97.21%).

**Fig. 1e:**
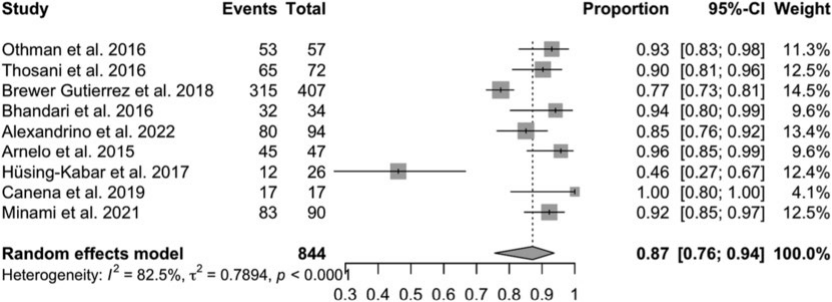
Forest plots summarizing pooled event rates and 95% confidence intervals for infectious adverse events following peroral cholangioscopy across the included studies. (V) Clinical success rate (pooled event rate, 87.13%).

### 3.4 Antibiotic Use and Subgroup Analysis

Antibiotic prophylaxis strategies varied substantially across the 12 included studies ( Table 3 ). Five studies (Bhandari et al., Alexandrino et al., Arnelo et al., Hüsing-Kabar et al., and Canena et al.) administered preprocedure or periprocedural antibiotics to all patients, most commonly with intravenous fluoroquinolones, third-generation cephalosporins, or piperacillin–tazobactam. Two studies (Othman et al. and Thosani et al.) deliberately excluded patients who had received prior antibiotics to evaluate procedure-related bacteremia, while five studies (Pereira et al., Brewer-Gutierrez et al., Chandan et al., Gustafsson et al., and Minami et al.) reported variable or unspecified prophylaxis practices. Among these, only Gustafsson et al. ( *n* = 1605) provided registry-level data on antibiotic use, with 81.4% of procedures performed under prophylaxis, yet no reduction in infectious complications was observed (3.4% vs. 3.7% in nonprophylaxis cases; *p* = not significant).

**Table 3:** Summarizing the antibiotic use (timing of administration, use, and agent) across the included studies.

No.	Study (first Author et al., year)	Population/procedure	Antibiotic prophylaxis (use and agent)	Timing of administration	Notes/key findings
1	**Othman 2016** ( *Gastrointest Endosc* )	ERCP with SOC; prospective bacteremia study	**None** —patients receiving antibiotics within 7 days were excluded	—(no preprocedure or postprocedure)	Observed bacteremia 27.8%; designed to assess infection risk without antibiotic use
2	**Pereira 2022** ( *Endosc Int Open* )	Digital SOC for indeterminate biliary strictures (AI study)	NR	NR	AI algorithm training dataset; infection outcomes not described
3	**Thosani 2016** ( *Endoscopy* )	ERCP + SOC; prospective bacteremia study	Single IV ciprofloxacin 400 mg given to all patients after SOC	**Postprocedure** (immediately after final blood draw)	No preprocedure AP; 9.7% required additional antibiotics for infection
4	**Chandan 2024** ( *Gastrointest Endosc* , MAUDE database)	Device adverse-event registry (SpyGlass DS/DS II)	Not applicable—passive database without clinical AP data	—	3.5% patient-related AEs; no prophylaxis information available
5	**Brewer-Gutierrez 2018** ( *Clin Gastroenterol Hepatol* )	International multicenter SOC for difficult stones	NR	NR	3.7% adverse events; no mention of antibiotic prophylaxis
6	**Bhandari 2016** ( *Gastrointest Endosc* )	SOC-guided laser lithotripsy for Mirizzi/cystic duct stones	NR	NR	Mild transient AEs (fever, pain, pancreatitis); no AP details
7	**Alexandrino 2022** ( *Dig Dis Sci* )	Multicenter SOC-guided lithotripsy for difficult stones	IV fluoroquinolone or third-generation cephalosporin in all patients	**Intraprocedural**	Uniform protocol; no severe infections reported
8	**Arnelo 2015** ( *Endoscopy* )	PSC patients undergoing SOC	NR	NR	No antibiotic data provided in manuscript
9	**Hüsing-Kabar 2017** ( *World J Gastroenterol* )	Liver-transplant recipients undergoing ERCP+SOC	Peri-interventional antibiotics (piperacillin/tazobactam common)	**Periprocedural**	Used in 92.8% of cases; 48.6% of isolates resistant to empiric AP
10	**Canena 2019** ( *GE Port J Gastroenterol* )	SOC-guided lithotripsy for difficult CBD stones	Single IV fluoroquinolone or third-generation cephalosporin for all	**Intraprocedural**	Standardized AP; no severe infectious AEs
11	**Gustafsson 2023** ( *Endosc Int Open* , GallRiks registry)	1,605 ERCP+SOC procedures in Sweden	AP recorded in 81.4% (type/dose not captured)	**Registry-level** , timing NR	No difference in infection rate with vs. without AP (3.4% vs. 3.7%)
12	**Minami 2021** ( *J Clin Med* )	Digital SOC for diagnosis and therapy	Antibiotic use at physician’s discretion; agent unspecified	**Some preprocedure** , nonstandardized	No preprocedure antibiotic predicted post-SOC fever ≥38 °C

Across studies without routine prophylaxis, the pooled bacteremia rate averaged 12.3% (range 0.2–27.8%), and cholangitis occurred in 5.2% (range 1.5–7.0%). In contrast, studies employing preprocedure or periprocedural antibiotics reported lower pooled bacteremia (7.2%, range 0.02–23.5%) and similar cholangitis (3.5%, range 0–8.2%) rates. Although prophylaxis appeared to modestly decrease bacteremia incidence numerically, this did not translate into a consistent reduction in clinically significant infection or cholangitis.

In prospective cohorts that systematically obtained blood cultures (Othman et al., Thosani et al., Hüsing-Kabar et al.), transient bacteremia was common but typically self-limited. Only a small subset developed symptomatic cholangitis or sepsis, suggesting that bacteremia alone may not represent a clinically meaningful endpoint. Importantly, studies using postprocedure-only prophylaxis (Thosani et al.) demonstrated bacteremia rates comparable to those with no prophylaxis (27.8% vs. 8.8%), indicating that delayed antibiotic administration may be ineffective. Conversely, Minami et al. found that absence of preprocedure antibiotics independently predicted postprocedural fever ( *p* < 0.01), supporting the role of timely preprocedural prophylaxis in reducing febrile complications.

## 4 Validation of Meta-analysis Results

### 4.1 Sensitivity Analysis

Multiple sensitivity analyses were performed to evaluate the robustness of the pooled bacteremia estimates and to explore potential sources of heterogeneity. Exclusion of large registry-level studies with sample sizes greater than 1000 resulted in a pooled bacteremia rate of 9.86% (95% CI, 2.71–30.02), with persistently high heterogeneity ( *I* ^2^ = 94.0%). Restriction to prospective studies alone yielded a higher pooled bacteremia rate of 21.09% (95% CI, 5.50–55.09), with substantial heterogeneity remaining ( *I* ^2^ = 85.0%). When the analysis was limited to therapeutic SOC procedures, the pooled bacteremia rate was 10.41% (95% CI, 2.19–37.60), with heterogeneity remaining high ( *I* ^2^ = 96.0%). In contrast, studies limited to lithotripsy-only procedures demonstrated a pooled bacteremia rate of 6.50% (95% CI, 0.21–69.56), again with high heterogeneity ( *I* ^2^ = 90.0%).

Further stratification by procedural indication demonstrated wide variability in bacteremia rates. Stone-related indications were associated with a pooled bacteremia rate of 6.50% (95% CI, 0.21–69.56), mixed indications with 10.65% (95% CI, 2.32–37.39), and other indications with 19.57% (95% CI, 0.00–100.00), all with substantial heterogeneity. A single study evaluating strictures reported a bacteremia rate of 1.18% (95% CI, 0.17–7.88), precluding assessment of heterogeneity. Across all sensitivity analyses, heterogeneity remained substantial, and no individual restriction materially altered the magnitude of the pooled bacteremia estimate (Supplementary **Tables 4 and 5** ).

Leave-one-out analyses demonstrated that omission of any single study resulted in pooled bacteremia rates ranging from approximately 5.0–9.0%, with *I* ^2^ values consistently exceeding 94%. Exploratory stratification by geographic region similarly demonstrated wide and overlapping CIs, with pooled bacteremia rates of 4.84% in studies from the United States, 11.04% in European studies, 11.43% in Asian studies, and 0.25% in multiregional cohorts. Owing to the small number of studies within each regional subgroup, these findings were considered descriptive and exploratory, and no formal comparisons were performed (Supplementary **Table 6** and Supplementary **Fig. 1C** ).

### 4.2 Meta-regression and Heterogeneity

To further explore sources of heterogeneity in bacteremia rates, univariable mixed-effects meta-regression analyses were conducted. Meta-regression by procedure type explained a limited proportion of between-study heterogeneity ( *R* ^2^ = 13.3%, where *R* ^2^ represents the proportion of between-study variance [τ ^2^ ] explained by the moderator) and did not identify procedure type as a statistically significant moderator of bacteremia rates ( *p* = 0.27). Substantial residual heterogeneity persisted following adjustment ( *I* ^2^ = 96.35%), indicating that differences in procedure type alone were insufficient to account for the observed variability across studies.

In contrast, meta-regression by study design demonstrated that prospective versus retrospective design was a statistically significant moderator of bacteremia rates ( *p* = 0.018). Study design accounted for approximately 43.8% of the between-study heterogeneity, with prospective studies reporting higher bacteremia rates compared with retrospective studies. Despite this reduction in heterogeneity, significant residual heterogeneity remained ( *I* ^2^ = 94.08%), suggesting that additional factors, including differences in surveillance intensity, bacteremia ascertainment methods, patient selection, and underlying disease severity, likely contribute to the remaining variability (Supplementary **Table 7** ).

### 4.3 Publication Bias

Assessment of small-study effects using Egger’s linear regression test did not demonstrate evidence of funnel plot asymmetry ( *t* -statistic [ *t* ] = 0.12, *p* = 0.90). The estimated intercept was small and not statistically significant (bias estimate = 0.32, SE = 2.56), suggesting no detectable association between study size and effect magnitude. Visual inspection of the funnel plot was consistent with these findings (56). The funnel plot is summarized in Supplementary **Fig. 1D** .

## 5 Discussion

SOC has become an essential tool in the management of complex biliary tract disorders, particularly difficult-to-remove stones, indeterminate strictures, and intraductal lesions and their biopsies. Our meta-analysis of 12 studies, comprising 4413 cholangioscopy-guided procedures, demonstrated high infectious complications, notably bacteremia (6.68%), cholangitis (4.59%), and pancreatitis (3.54%). High technical (97.21%) and clinical (87.13%) success rates were also noted, supporting its reliability and efficacy. Mild AEs predominated, but severe and fatal complications were rare. Importantly, antibiotic prophylaxis did not consistently prevent infection, likely due to variability in timing, selection, and patient risk factors.

SOC offers several advantages over traditional ERCP, such as improved maneuverability, better visualization, and direct stone treatment, increasing yield of intraductal biopsies, and reducing the need for repeat procedures. 17 However, the risk of bacteremia, often due to biliary instrumentation, remains significant, particularly when biopsies are performed or when preprocedural antibiotics are not administered. Infection rates associated with conventional ERCP without SOC are generally lower, with bacteremia reported in approximately **2–5%** of procedures and post-ERCP cholangitis occurring in **0.5–3%** of cases in prospective studies. The higher rates observed in SOC procedures may reflect additional biliary instrumentation, longer procedural times, and intraductal irrigation required during SOC. In addition, because most studies included in this meta-analysis did not systematically obtain preprocedural blood cultures, the possibility that bacteremia was present prior to ERCP cannot be completely excluded. Despite widespread antibiotic prophylaxis, its role in preventing postprocedural infections, especially cholangitis, remains uncertain. 18

SOC may elevate the risk of bacteremia through several procedural- and patient-related mechanisms. Disruption of the biliary epithelium, increased intraductal pressure from irrigation, and retrograde bacterial translocation all contribute to infectious complications. 19 The use of continuous irrigation with saline, water, or carbon dioxide to enhance visualization can lead to elevated intrabiliary pressures, promoting bacterial entry into the bloodstream. Furthermore, turbulence generated during interventions, such as electrohydraulic lithotripsy or biopsies, may exacerbate mucosal trauma and bacterial dissemination. 20 While transient bacteremia is often clinically silent, it poses a significant risk in high-risk populations, including patients with biliary obstruction, primary sclerosing cholangitis, immunosuppression, prior biliary stent placement, and advanced age. 21 These factors, coupled with the inherently therapeutic nature of SOC, underscore the importance of judicious patient selection and consideration of prophylactic strategies in those most vulnerable to postprocedural infections. 5 Conflicting data exist regarding the use of antibiotic prophylaxis in cholangioscopic procedures, as studies by Gustafsson and Minami showed no clear benefit of antibiotic prophylaxis in reducing overall AEs, highlighting the need for more targeted, risk-based approaches. ^7,22^ Our findings highlight that *when* antibiotics are given may be as critical as *whether* they are used. Studies employing preprocedure or periprocedural antibiotics (Canena et al., Alexandrino et al., Hüsing-Kabar et al.) reported lower cholangitis rates than those giving antibiotics only after SOC (Thosani et al.) or none at all (Othman et al.). Fluoroquinolones and third-generation cephalosporins were most frequently used. Registry data (Gustafsson et al.) further suggest that routine prophylaxis alone may not reduce infection risk without addressing drainage completeness. Transient bacteremia, particularly in studies in which routine postprocedure cultures were obtained, often lacked clinical relevance. Only a minority of patients developed symptomatic infection, supporting the notion that bacteremia alone should not be equated with infectious complications.

Given the increasing utilization of SOC, future directions should focus on standardizing infection prevention protocols and identifying patient subsets who would most benefit from antibiotic prophylaxis. Randomized controlled trials comparing electrohydraulic lithotripsy and laser lithotripsy, as well as trials stratifying outcomes based on antibiotic strategy, are warranted. Furthermore, establishing a risk score to predict infectious complications based on procedural and patient-specific factors may help guide prophylactic decision-making. 23 24 Integration of advanced imaging modalities and artificial intelligence may further enhance cholangioscopy’s diagnostic yield while minimizing procedural time and complication rates. 25 Also, while cholangioscopy offers clear clinical advantages in the management of difficult biliary stones and strictures, its widespread adoption raises important questions regarding cost-effectiveness. Compared to conventional ERCP techniques, cholangioscopy requires additional equipment, longer procedure times, and greater technical expertise, all contributing to increased procedural costs. In randomized controlled trials, including the study by Bang et al., cholangioscopy was associated with higher mean procedural costs (e.g., $16,684 vs. $10,626 for balloon sphincteroplasty), although the difference did not reach statistical significance, likely due to sample size limitations. 26 However, cholangioscopy may reduce the need for repeat procedures, hospital readmissions, and ancillary imaging, which could offset the initial cost. Moreover, higher single-session ductal clearance rates, particularly with laser lithotripsy, may enhance overall healthcare efficiency. From a health economics perspective, cost-effectiveness analyses incorporating not only direct procedural costs but also downstream outcomes, such as treatment durability, complication rates, and quality-adjusted life years are needed to guide appropriate resource allocation. 27

This study has some limitations, including significant heterogeneity in patient populations, procedural details, and outcome definitions. First, substantial statistical heterogeneity was observed across multiple outcomes, particularly for bacteremia where the *I* ^2^ statistic exceeded 90%. Such high heterogeneity limits the reliability and interpretability of pooled estimates and suggests that differences in study populations, methodologies, and outcome assessment may significantly influence the results. Second, the definition and detection of bacteremia varied considerably among the included studies. Differences in blood culture protocols, timing of sampling, and surveillance intensity introduce the potential for detection bias and may partly explain the variability in reported bacteremia rates. Third, most included studies were observational, which increases the risk of selection bias, confounding, and publication bias. Additionally, the included cohorts were clinically heterogeneous, encompassing patients with PSC, liver transplantation, and altered anatomy. Although sensitivity and subgroup analyses were performed, residual confounding cannot be fully excluded.

This study has several strengths. It represents the most comprehensive synthesis to date of infectious complications following SOC, with a specific focus on bacteremia as a clinically relevant outcome. Strengths include a rigorous and transparent methodology conducted in accordance with PRISMA guidelines, inclusion of diverse study designs and geographic regions, and standardized AE classification using the ASGE lexicon. The analysis incorporated extensive sensitivity analyses, leave-one-out assessments, and exploratory meta-regression to systematically evaluate sources of heterogeneity rather than relying solely on pooled estimates. Additionally, the inclusion of registry-level data alongside prospective cohorts provides a broad perspective on real-world practice and outcomes. Formal assessment of publication bias further strengthens the validity of the findings.

In conclusion, SOC was associated with appreciable rates of bacteremia, cholangitis, and pancreatitis, despite high technical and clinical success rates. Infectious complications were common but predominantly mild, whereas severe and fatal AEs were rare. Marked heterogeneity across studies highlights the influence of study design, procedural complexity, and infection-surveillance practices, with prospective studies reporting higher bacteremia rates. The available evidence suggests that the clinical significance of bacteremia following SOC remains uncertain and may depend on patient risk factors and procedural complexity. Future prospective studies are needed to standardize outcome definitions, clarify the clinical significance of transient bacteremia, and develop risk-based strategies to guide antibiotic use in patients undergoing SOC.

## Supplementary Material

Ergänzendes Material Supplementary Material
