# Supplementary material for: Infectious Complications and Safety Outcomes in Cholangioscopy: A Systematic Review and Meta-Analysis
Source: Endosc Int Open. 2026 Jun 10;14:a28779645. doi: 10.1055/a-2877-9645 (PMC13289773; doi:10.1055/a-2877-9645)
Supplement: Supplementary file 1 — Ergänzendes Material [file 10-1055-a-2877-9645_28823161.pdf]

Supplementary Material: Infectious Complications and Safety Outcomes in Cholangioscopy: A Systematic Review and Meta-Analysis

Shivangini Duggal MD, Akshay Sharma MD, Vishali Moond MD, Simran Joshi MD, Mehak Sachdeva MD, Nirav Thosani MD, MHA, Prateek Harne MD

Section	Item	PRISMA Checklist Item	Location in Manuscript
**TITLE**	1	Identify the report as a systematic review and/or meta-analysis	Title page
**ABSTRACT**	2	Provide a structured abstract including background, methods, results, and conclusions	Abstract
**INTRODUCTION**	3	Describe the rationale for the review in the context of existing knowledge	Introduction
	4	Provide explicit statement of objectives or research questions	Introduction
**METHODS**	5	Specify inclusion and exclusion criteria and how studies were grouped	Methods – Study Selection
	6	Specify all information sources and date last searched	Methods – Search Strategy
	7	Present full search strategy for at least one database	Supplementary Material
	8	Describe study selection process	Methods – Study Selection
	9	Describe data collection process	Methods – Data Abstraction
	10	List and define all outcomes for which data were sought	Methods – Outcomes Assessed
	11	Describe risk-of-bias assessment methods	Methods – Risk-of-Bias Assessment
	12	Specify effect measures used in synthesis	Methods – Statistical Analysis
	13	Describe synthesis methods including meta-analysis models	Methods – Statistical Analysis
	14	Describe methods used to explore heterogeneity	Methods – Sensitivity Analysis & Meta-regression
	15	Describe methods used to assess reporting bias	Methods – Publication Bias
	16	Describe additional analyses (e.g., subgroup, sensitivity)	Methods – Statistical Analysis

**RESULTS**	17	Provide number of records identified, screened, included, excluded	Results – Search Strategy (PRISMA flow diagram)
	18	Present characteristics of included studies	Table 1
	19	Present risk-of-bias assessments	Supplementary Table
	20	Present results of individual studies	Results section
	21	Present results of syntheses	Results – Meta-analysis outcomes
	22	Present results of heterogeneity analyses	Results – Sensitivity analysis & meta-regression
	23	Present results of publication bias assessment	Results – Publication bias
**DISCUSSION**	24	Provide summary of evidence	Discussion
	25	Discuss limitations of included evidence and review process	Discussion – Limitations
	26	Discuss implications for practice and research	Discussion
**OTHER INFORMATION**	27	Provide registration and protocol information	Methods
	28	Describe sources of funding	Financial disclosure
	29	Declare competing interests	Financial disclosure
	30	Describe availability of data and materials	Methods / Supplementary

Supplementary Table 1. PRISMA checklist for this meta-analysis.

Database	Search Strategy	Filters / Limits	Date of Search
**PubMed**	("cholangioscopy" OR "single-operator cholangioscopy" OR "peroral cholangioscopy" OR "SpyGlass" OR "SpyGlass DS" OR "SpyGlass DS II") AND ("bacteremia" OR "bacteraemia" OR "cholangitis" OR "infection" OR "sepsis" OR "adverse events" OR "complications")	Human studies; English language	March 2025
**Embase**	('cholangioscopy' OR 'single operator cholangioscopy' OR 'peroral cholangioscopy' OR 'SpyGlass') AND ('bacteremia' OR 'bacteraemia' OR 'cholangitis' OR 'infection' OR 'sepsis' OR 'complication' OR 'adverse event')	Human studies; English language	March 2025
**Scopus**	TITLE-ABS-KEY ("cholangioscopy" OR "single operator cholangioscopy" OR "peroral cholangioscopy" OR "SpyGlass") AND TITLE-ABS-KEY ("bacteremia" OR "bacteraemia" OR "cholangitis" OR "infection" OR "sepsis" OR "adverse events" OR "complications")	English language	March 2025
**Cochrane Library**	("cholangioscopy" OR "SpyGlass" OR "peroral cholangioscopy" OR "single operator cholangioscopy") AND ("bacteremia" OR "cholangitis" OR "infection" OR "complications")	Trials and observational studies	March 2025
**Google Scholar**	"cholangioscopy" OR "SpyGlass cholangioscopy" AND "bacteremia" OR "cholangitis" OR "infection" OR "complications"	First 200 most relevant results screened	March 2025

Supplementary Table 2. Search Strategies for this meta-analysis.

	SELECTION				COMPARABILITY	OUTCOME			SCORE	QUALITY
	Representativeness of the average adult in community	Cohort size	Information on clinical outcomes	Outcome not present at start	Factors comparable between the groups	Adequate clinical assessment	Follow up time	Adequacy of follow-up	MAX=8	HIGH>6, MEDIUM 4 to 6, LOW <4
	Population based: 1; Multi-center: 0.5; Single-center: 0	>40 patients: 1; 39 to 20: 0.5; <20: 0	Information with clarity: 1; Information derived from percentage value: 0.5; unclear: 0	not present: 1; present: 0	yes: 1; no: 0	yes: 1; no: 0	yes: 1; not mentioned: 0	All patients followed up: 1; >50% followed up: 0.5; <50% followed up OR not mentioned: 0		
Othman et al.	0	1	1	1	1	1	1	1	7	High
Pereira et al.	0	1	1	1	1	1	1	1	7	High
Thosani et al.	0.5	1	1	1	1	1	1	1	7.5	High
Chandan et al.	0	1	0.5	1	0	0	0	0.5	3	Low
Brewer Gutierrez et al.	0.5	1	1	1	1	1	1	0.5	7	High
Bhandari et al.	0	0.5	1	1	0	1	1	1	5.5	Medium
Alexandrino et al.	0.5	1	1	1	1	1	1	1	7.5	High
Arnelo et al.	0	0.5	1	1	0	1	1	1	5.5	Medium
Hüsing-Kabar et al.	0	0	1	1	0	1	1	1	5	Medium
Canena et al.	0.5	0	1	1	0	1	1	1	5.5	Medium
Gustafsson et al.	1	1	1	1	1	1	1	1	8	High
Minami et al.	0	1	1	1	0	1	1	1	6	Medium

Supplementary Table 3. This table summarizes detailed quality assessment of included studies. An 8-point rubric spanning three domains: Selection (representativeness of source population, cohort size, clarity of outcome information, and confirmation that the outcome was absent at baseline), Comparability (between-group factor balance/adjustment), and Outcome (adequacy of clinical assessment, reporting of follow-up duration, and adequacy of follow-up), was used.

Sensitivity Analysis	N	Pooled Rate (%)	95% CI	I² (%)
1. Excluding studies with N > 1000	10	9.86	2.71 – 30.02	94.0
**2. Prospective studies only**	**6**	**21.09**	**5.50 – 55.09**	**85.0**
3. Sensitivity Analysis by Procedure type				
Therapeutic SOC only	7	10.41	2.19 – 37.60	96.0
Lithotripsy only	4	6.50	0.21 – 69.56	90.0
4. Sensitivity Analysis by Indication				
Stones indication (0)	4	6.50	0.21 – 69.56	90.0
Strictures indication (1)	1	1.18	0.17 – 7.88	NA
Mixed indication (2)	4	10.65	2.32 – 37.39	96.0
Other indication (3)	2	19.57	0.00 – 100.00	90.0

Supplementary Table 4. Sensitivity analyses were performed to evaluate the robustness of the pooled bacteremia estimate and to explore potential sources of heterogeneity. Analyses included exclusion of large registry-level studies (N > 1000), restriction to prospective studies, stratification by procedure type, and stratification by procedural indication. Pooled estimates were generated using random-effects models. Heterogeneity remained substantial across most sensitivity analyses, reflecting persistent between-study variability.

Region	Studies (k)	Pooled bacteremia (%)	95% CI
USA	3	4.84	0.01–95.46
Europe	6	11.04	1.53–49.83
Asia	2	11.43	0.10–94.45
Multiregional	1	0.25	0.03–1.72

Supplementary Table 5. Pooled bacteremia rates were estimated using random-effects models and stratified by study region. Confidence intervals were wide and overlapping across regions, reflecting the small number of studies within each subgroup and substantial between-study heterogeneity. These analyses are exploratory and descriptive and were not intended for formal regional comparisons.

Thieme

Omitted Study	Pooled Rate (%)	95% CI	I^2 (%)
Othman 2016	6.46	1.64 – 22.18	95.9
Pereira 2022	7.59	2.06 – 24.24	95.8
Thosani 2016	5.71	1.52 – 19.20	95.2
**Chandan 2024**	**8.96**	2.79 – 25.22	94.4
Brewer-Gutierrez 2018	8.57	2.67 – 24.27	95.7
Bhandari 2016	6.70	1.72 – 22.83	95.9
Alexandrino 2022	5.85	1.53 – 19.89	95.4
Arnelo 2015	7.25	1.91 – 23.84	95.9
**Hüsing-Kabar 2017**	**5.02**	1.60 – 14.67	94.7
Canena 2019	5.87	1.55 – 19.86	95.8
Gustafsson 2023	7.08	1.82 – 23.85	94.2
Minami 2021	6.16	1.58 – 21.21	95.7
**Overall (none omitted)**	**6.68**	1.96 – 20.44	**95.5**

Supplementary Table 6. Leave-one-out sensitivity analysis of pooled bacteremia rates following SOC. Each study was sequentially omitted to assess its influence on the pooled bacteremia estimate. Pooled rates and heterogeneity remained largely stable across iterations, indicating that no single study disproportionately influenced the overall estimate. Persistently high I^2 values reflect substantial residual heterogeneity across studies.

.

Moderator	Comparison	Coefficient (logit)	95% CI	P value	R^2 (%)
Study design	Prospective vs Retrospective	2.55	0.54–4.56	0.018	43.8
Procedure type	Therapeutic SOC vs Reference	0.47	−2.27–3.21	0.71	13.3

Supplementary Table 7. Univariable meta-regression analyses evaluating moderators of bacteremia rates following SOC. Exploratory mixed-effects meta-regression was performed to assess the association between study-level moderators and bacteremia rates. Study design emerged as a statistically significant moderator, with prospective studies reporting higher bacteremia rates than retrospective studies, accounting for 43.8% of between-study heterogeneity. Procedure type was not a significant moderator and explained a limited proportion of heterogeneity. Meta-regression analyses were exploratory and hypothesis-generating.

REFERENCES

1. Page, Matthew J., Joanne E. McKenzie, Patrick M. Bossuyt, Isabelle Boutron, Tammy C. Hoffmann, Cynthia D. Mulrow, Larissa Shamseer et al. "The PRISMA 2020 statement: an updated guideline for reporting systematic reviews." *bmj* 372 (2021).
